# Disaster Medicine Core Competencies: Comparative Analysis of Emergency Medicine Residency Training in Taiwan and the United States

**DOI:** 10.5811/westjem.24961

**Published:** 2025-06-25

**Authors:** Joyce Tay, Wei-Kuo Chou, Ming-Tai Cheng, Chih-Wei Yang, Shuo-Kuen Huang, Chien-Hao Lin

**Affiliations:** * National Taiwan University Hospital, Department of Emergency Medicine, Taipei, Taiwan; † National Taiwan University College of Medicine, Department of Medical Education & Bioethics, Taipei, Taiwan; ‡ National Taiwan University Hospital, Department of Medical Education, Taipei, Taiwan; § National Taiwan University Hospital Hsin-Chu Branch, Department of Emergency Medicine, Hsinchu, Taiwan

## Abstract

**Background:**

Situated in the western Pacific Ocean, Taiwan has faced a diverse array of natural and man-made disasters. Since 2000, disaster medicine education has been progressively integrated into various medical professions, with a focus on training disaster medical assistance teams, managing chemical and radiological emergencies, and enhancing prehospital and hospital emergency management capabilities. Despite the key roles of emergency physicians (EP) as primary responders and crucial managerial personnel during disasters, a comprehensive assessment of the disaster medicine core competencies (DMCC) required for emergency medicine (EM) residency training might serve as a blueprint for Taiwan’s EM residency core curriculum. We sought to survey the most critical DMCCs, prioritize them, and determine their appropriateness for the EM residency training program. We also compare dthe prioritization of DMCCs between Taiwan and the United States.

**Methods:**

To accomplish these objectives, we employed a modified Delphi method over three rounds. Initially, three EPs developed a draft of DMCCs for Taiwan. This draft, including 42 DMCCs, was subsequently reviewed by a task force comprising 22 leaders in disaster medicine from EM residency training hospitals across Taiwan. The Delphi method facilitated consensus on the DMCCs through three iterative rounds of polling, with each round evaluating the appropriateness of the proposed competencies. The study also compared the prioritized DMCCs proposed in both Taiwan and the US.

**Results:**

The following 15 DMCCs were rated as highly appropriate with high consensus agreement: personal protective equipment (PPE); decontamination; incident command systems; mass casualty incidents; basic concepts and nomenclature of disaster medicine; medical response to chemical emergencies; triage; identification, notification, activation, and information collection; medical response to radiation emergencies; medical response to bioterrorism and biological emergencies; mental health; disaster exercises; prehospital disaster management; communication and information management; and health consequences of different disasters. A comparison with DMCCs in the US revealed shared prioritization for PPE and decontamination competencies. However, Taiwan placed greater emphasis on prehospital disaster operation management, mental health implications, and health consequences across different disasters, while the US focused more extensively on emergency management within hospitals.

**Conclusion:**

The expert-consensus-driven ranking of DMCCs in the study showed noteworthy agreement with the US. However, the roles of EPs, experience of previous disasters, and government policies may influence specific competencies. This underscores the importance of incorporating local context into disaster medicine training.

## INTRODUCTION

Taiwan is an industrialized island with a population of approximately 23.4 million, located in the subtropical Pacific Ocean of Southeast Asia. According to the World Bank, more than 73% of Taiwan’s land area and population are exposed to three or more natural disasters annually.^1^ Situated within the Pacific “Ring of Fire,” Taiwan is prone to frequent earthquakes. The 1999 Chi-Chi Earthquake was the most devastating to date, resulting in 2,347 fatalities, 8,722 injuries, and estimated property damage exceeding 92 billion US dollars.^2^ This catastrophic event prompted the government and society to prioritize disaster management, including the development of disaster medicine (DM) education.

Taiwan lies along the primary typhoon strike path in the Northwest Pacific region. Typhoons Nari in 2001^3^ and Morakot^4^ were among the most severe to hit the island, causing extensive damage and loss of life. Furthermore, the rapid growth of international transportation has facilitated the transmission of infectious diseases such as severe acute respiratory syndrome,^5^ H1N1 influenza,^6^ and coronavirus disease 2019 to the island.^7^ These emerging infectious diseases have spread globally through large-scale transmission and pose significant challenges to communities worldwide. Over the past several decades, with rapid advancements in technology, manufacturing, and transportation, technical disasters such as fires and hazardous material accidents have also become increasingly prevalent in Taiwan. Moreover, the country currently faces a significant risk of potential military conflicts.^8^

Consequently, the Taiwanese government and various healthcare stakeholders have been actively and continuously developing DM education within the country, emphasizing the ability of medical personnel to respond to various types of disasters and emergencies. In Taiwan, emergency physicians (EP) play critical roles as frontline disaster responders before, during, and after disasters. They actively participate in the development and execution of disaster preparedness and response plans for medical and healthcare-related emergencies. During disasters, they manage mass casualties in both prehospital settings and emergency departments by coordinating triage, treatment, and casualty transfers. After disasters, EPs also engage in relief and recovery efforts, assisting in rehabilitation and rebuilding the healthcare system. As a result, DM education is particularly emphasized for emergency medicine (EM) residents. However, despite the diverse goals of DM, the core competencies for EM residency training in Taiwan have not yet been fully standardized.^9^

In the interdisciplinary context of DM education, establishing core competencies is particularly crucial.^10^ Effectively addressing various natural disasters, man-made disaster events, and public health crises requires knowledge and skills spanning various professional domains. Clear definitions of core competencies can ensure that medical students and residents possess the necessary skills to prepare for disasters. Core competencies are currently being developed in the context of DM education, both internationally and in the United States.^11^–^13^

**Population Health Research Capsule**

What do we already know about this issue?

*Disaster medicine core competencies (DMCC) are essential for training emergency medicine (EM) residents, but they vary by region and healthcare system*.

What was the research question?

*What are the most critical DMCCs for EM residencies in Taiwan compared to the US?*

What was the major inding of the study?

*The top five DMCCs were personal protective equipment (4.8); decontamination (4.7); incident management system (4.7); mass casualty incidents (4.6); and disaster medicine basics (4.6)*.

How does this improve population health?

*Developing EM residency training based on DMCCs aligned with local disaster response needs is essential for strengthening healthcare system resilience*.

Establishing these core competencies is essential to ensuring comprehensive coverage and continuous improvement of education and training. By defining clear learning objectives and assessment criteria, adaptability and resilience can be cultivated in future medical professionals in order to better address challenges related to future disaster events.^7^ Although the types of disasters in different areas may be similar, differences in geographical environments, cultures, disaster response mechanisms, and government systems necessitate regional variations in DM core competencies (DMCC). The adoption of “competency-based medical education” by the Accreditation Council for Graduate Medical Education (ACGME) in the US highlights a systems-based practice^14^ that applies to DM as well. Comparative research on this topic is currently lacking in the literature.

Since disaster medicine covers a wide range of capabilities and skills, it is necessary to address the most critical DMCCs and include them in EM residency training. In this study we aimed to investigate the prioritized DMCCs for EM residency training during the limited EM training period in Taiwan. Subsequently, we compared these to the established practices in the US to evaluate the potential variations that may have arisen as a result of the different political or regional backgrounds of these two jurisdictions.

## METHODS

In 2023, a modified three-round Delphi method was used to formulate the recommended DMCCs for EM residency training in Taiwan. This study was reviewed and approved by the Research Ethics Committee D of the National Taiwan University Hospital (NTUH-REC No. 202207185W). Initially, three EPs serving as senior DM trainers systematically reviewed the relevant literature, including DMCCs for EM residency training in the US^12^ and other publications addressing disaster medicine, competencies, and emergencies and disasters on PubMed and Web of Science,^11^,^13^ so that potential elements of DMCC that would be later evaluated by the full panel of 22 experts would not omit any important topics for further consideration. Acknowledging the variations in governmental systems, healthcare infrastructures, and cultural contexts across nations and healthcare professions that could influence DMCCs, a Chinese version of the DMCCs, along with meticulously drafted detailed objectives, were subsequently developed and tailored specifically for the Taiwanese context. All three EPs agreed with the modifications and final draft.

A task force was established for this study, which included a total of 22 experts from February–October 2023. The task force comprised 20 members of the Disaster Response Committee of the Taiwan Society of Emergency Medicine and two senior DM trainers from the Taiwan Emergency Management Association. The participants of the task force all received specialized training in various subfields of disaster medicine—such as radiation incidents, chemical incidents, disaster medical assistance teams (DMAT), and hospital emergency management—after their residency and subsequently served as trainers in disaster medicine. They had a mean duration of professional experience of 12.8 years (SD 6.2) in the field of DM, and they were all DM program managers in EM residency training hospitals across Taiwan. Sixteen of the participants were employed at medical centers, while the remaining six practiced at regional hospitals. They were also senior leaders of the DMATs. Among the three EPs who initiated the draft, they have published five, eight, and nine articles in peer-reviewed English-language journals, respectively. Regarding the remaining 19 experts, they have collectively published 47 articles related to DM. Furthermore, at least 12 of these experts have authored at least one article in the DM literature.

Our primary goal was to identify and prioritize the most critical DMCCs and assess their appropriateness for the EM residency training program in Taiwan. To achieve consensus among the task force participants, we employed the modified Delphi method. This entailed three iterative rounds of polling the participants to characterize their initial degree of consensus. In each round, the participants rated the appropriateness of each DMCC on a five-point Likert scale (1 = very inappropriate, 2 = inappropriate, 3 = fair, 4 = appropriate, and 5 = very appropriate). Consensus determination was contingent on a high level of agreement after three rounds, defined as an interquartile range (IQR) of ≤1 and <2 participants changing scores between the final two rounds. Competencies without consensus were not deleted after the first round but were carried forward to the next rounds until a consensus was reached. The participants also revised the wording of the objectives for each DMCC. The experts were provided with feedback from all participants in the previous round to inform their decisions and aid in the establishment of a consensus during the second round. Based on expert input, modifications were made where necessary, which ultimately led to improved consensus. The Delphi panel did not merely re-rate the competencies; instead, adjustments were considered when substantial feedback suggested necessary refinements. The median score for each DMCC was used to rank its appropriateness.

The secondary goal of this study was to compare the prioritization of DMCCs between Taiwan and the US. To enhance the ranking and facilitate comparison with US results, we used mean scores to determine the priority order when DMCCs had the same median scores. The criteria (median and mean scores ≥4, and IQR of ≤1) for selecting the most appropriate DMCCs were determined a priori, before conducting the Delphi rounds.

The ACGME core competencies serve as a fundamental framework for EM residency training programs in Taiwan. This study also mapped the DMCCs to these six core competencies to ensure their integration into the overall development of EM residency training. A panel of five experts, comprising three EPs and two medical education experts, was convened for this purpose. Each DMCC was systematically mapped to the six core competencies: patient care, medical knowledge, practice-based learning and improvement, interpersonal and communication skills, professionalism, and system-based practice. The experts independently assessed the correlations between each DMCC and the six core competencies. If more than four of the five experts agreed on the correlation, the DMCCs were categorized under the six core competencies of medical education. This threshold was established based on expert panel discussions to ensure that only competencies with strong consensus were classified as high agreement.

## RESULTS

Applying the modified Delphi method, the task force completed three iterative rounds of polling to investigate DMCC-related consensus. As a result, 42 DMCCs were evaluated during the third round. In the final round, a consensus was reached regarding 34 DMCCs. Fifteen of these exhibited mean scores of ≥4, indicating high appropriateness. These were as follows: personal protective equipment; decontamination; incident management system; mass casualty incidents; basic concepts and nomenclature of DM; medical response to chemical emergencies; triage; identification, notification, activation, and information collection; medical response to radiation emergencies; medical response to bioterrorism and biological emergencies; mental health; disaster exercises; prehospital disaster management; communication and information management; and health consequences of different disasters (Table 1).

The detailed objectives of each DMCC form the main context of the core competencies. The detailed objectives of the 15 DMCCs with high agreement and appropriateness listed in Table 2 were considered to form the context of disaster medicine training for EM residency. All of the DMCCs and their detailed objectives are presented in Appendix 1. The 42 DMCCs were mapped to the six core competencies defined by the ACGME for competency-based medical education. In cases where ≥4 raters among the five experts reached a consensus on the match, the DMCC was deemed to have been mapped to the selected core competencies (Table 3 and Appendix 2). Six of the 15 DMCCs with high levels of appropriateness were mapped to patient care, 12 to medical knowledge, one to interpersonal and communication skills, and seven to systems-based practice. None were determined to map to practice-based learning and improvement or professionalism. Eight DMCCs were mapped to two or more ACGME core competencies, and three DMCCs were mapped to three core competencies. The study compared the DMCCs in Taiwan to the established core competencies in the US to evaluate potential variations that may have arisen as a result of the different political or regional backgrounds of the two jurisdictions, as is summarized in Table 4.^12^

## DISCUSSION

The 15 DMCCs with high levels of agreement, and those deemed highly appropriate for EM residency training in Taiwan, primarily consisted of two main aspects. First, they encompassed the fundamental knowledge of DM, which integrates emergency management and EM into a multidisciplinary specialty. It is crucial for EPs to develop their domain knowledge and acquire essential skills based on the “all-hazards approach,” which applies to a wide range of disaster scenarios. Second, they must be proficient in response strategies and specialized skills for specific hazards, such as radiation, biological, and chemical incidents since EPs often serve as first responders within communities and healthcare systems when these types of hazards impact public health. These hazards present unique challenges in terms of response processes—requiring not only special medical care but also the protection of responders and facilities. Mishandling these hazards can exacerbate the situation and lead to further damage.

Nowadays, the DM training curriculum for EM residency in Taiwan consists of two main components, aligning with the two domains of DMCCs. Fundamental disaster medicine knowledge, including emergency command systems, disaster response frameworks, legislation, logistics, and public health, is delivered through an online multimedia program. Meanwhile, training for special incidents, such as chemical, radiological, and biological hazards, as well as hospital emergencies, combines online learning with real-world group simulations. However, we identified gaps in the current training, particularly in mental health, communication, and information management, which are rarely addressed. Additionally, we found that both hospital-based mass casualty incident (MCI) and prehospital disaster management should be equally emphasized, although this is not yet a universally accepted practice in Taiwan. Moreover, disaster exercises were highlighted in our findings but are not yet incorporated into the current curriculum. Our study provides clear objectives for future training design. To address these gaps, we plan to integrate mental health and communication training and develop new simulated scenarios for both prehospital and hospital MCI response to enhance practical disaster preparedness. Additionally, specific training for disaster exercises is still under development, requiring further refinement based on current consensus.

However, we acknowledge the challenges inherent in promoting the prioritized DMCCs according to our findings, in that time for residency training is limited. Spending time teaching these DMCCs to residents would necessarily require removing content in other areas. It is beyond the scope of this paper to determine what other topics might be sacrificed to include additional DMCC content. One potential approach to addressing this challenge is leveraging multimedia training. With the advancement of online education, some knowledge-based DMCCs, such as the fundamental concepts and nomenclature of DM, are now effectively delivered through online training programs. Meanwhile, DMCCs ranked as relatively lower priority, such as hospital emergency management, are considered more advanced competencies and are increasingly being integrated into DM subspecialty training.

The commonalities in EM residency training between Taiwan and the US suggest potentially universal elements across diverse countries, emphasizing considerations beyond governmental or cultural differences. However, some key differences were identified as well. In Taiwan, training for EPs places significant emphasis on prehospital disaster management, mental health, and the health-related consequences of different disasters—likely influenced by experiences and related disaster response strategies following events such as the Chi-Chi earthquake and various typhoons. During emergencies in Taiwan, when onsite medical assistance is required, EPs often serve as first responders dispatched to the scene, in a practice that mirrors that of Japan.^15^ Consequently, training for EM residents frequently includes disaster medical assistance team training and exercises conducted in out-of-hospital settings. The EPs also work closely with the emergency medical service (EMS) system and serve as primary responders in prehospital settings. Many EPs in Taiwan also hold roles as medical directors within the EMS system, providing medical direction in real time. Therefore, EPs often represent the best adjuncts to prehospital medical responses during disasters. This unique disaster response system may have influenced the differences observed in the DMCCs of the EM resident training programs between Taiwan and the US. Consequently, when developing DMCCs for specialty training, factors such as governmental policies and regulations, roles within the healthcare system, and previous disaster experiences should all be considered.

Additionally, when mapping the DMCCs for EM residency training in our study with the six core ACGME competencies we observed that, beyond patient care and medical knowledge, the third most significant domain of the six core competencies was systems-based practice. This further reinforced our earlier observations regarding the importance of understanding the disaster response systems implemented by both community-level and governmental authorities. Although DMCCs were similar in Taiwan and the US, the detailed mechanisms and operational procedures, such as incident management systems, may vary significantly between countries. Therefore, the concept of systems-based practice plays a crucial role in program development, ensuring that training aligns with each country’s specific disaster response framework and healthcare system.

It was also observed that there were no DMCCs mapped to practice-based learning and improvement among the top 15 DMCCs. Practice-based learning and improvement involves physicians’ abilities to engage in lifelong learning and improvement by systematically analyzing their practice and incorporating new evidence to further enhance patient care.^14^ Owing to the unpredictable and unprecedented nature of disasters, practical experience and scientific evidence specific to these situations remain limited. Therefore, learning from disaster-response experiences both nationally and internationally can significantly enhance DM training.

## LIMITATIONS

This study was subject to several key limitations worth noting. First, the single-country nature of the study may limit its generalizability, although certain identified commonalities between Taiwanese and US EM residency education suggest potentially universal elements that transcend governmental or cultural differences. Second, since the initial draft was created by three EPs and not the whole task force, there might have been some unconscious bias during the drafting process. However, the three senior DM trainers were asked to minimally change the concepts in the original document^12^ and only make the necessary changes for the context. All 22 participants were asked to revise the wording of the titles and objectives for each DMCC if they regarded them as unclear. The experts could also provide quantitative and qualitative feedback to aid in establishing a consensus during each round to minimize the potential bias from the initial drafting process.

Third, a formal Hazard Identification and Threat Assessment was incorporated into the development of our DMCCs, which may vary across different countries. However, the current DMCCs were reviewed by all participants, ensuring that all potential hazards and threats relevant to Taiwan were considered. Additionally, during the modified Delphi process, participants had the opportunity to propose additional competencies related to specific hazards. Ultimately, the current set of DMCCs likely provides a comprehensive consideration of potential hazards with the proviso that emerging external threats are continually evolving worldwide.^16^ Many countries have already expanded their training programs for various medical professions to include tactical medicine; however, a general consensus on this subject is still forthcoming, which may somewhat under-represent its significance. Regular reviews based on hazard identification and threat assessments and adjustments to governmental policies and global scenarios are, therefore, crucial to the field.

## CONCLUSION

The 15 expert- and consensus-driven disaster medicine core competencies in this report serve as a blueprint for EM residency training in Taiwan, emphasizing fundamental disaster knowledge and specialized response skills. While the current curriculum aligns with these domains, gaps remain in mental health, communication, and disaster exercises, and mass casualty incident-training with focus on both hospital and prehospital settings. Although Taiwan and the US share common DMCCs, Taiwan places greater emphasis on prehospital disaster management, mental health, and disaster-related health impacts, shaped by past disaster experiences. These differences underscore the need to consider governmental policies, healthcare roles, and disaster response systems when developing DMCCs for EM residency training, ensuring EPs are better prepared to respond to disasters within their local context.

## Supplementary Information



## Figures and Tables

**Table 1:** Final ranking of the disaster medicine core competencies for emergency medicine residency training in Taiwan.

Ranking	Competency	Median	Mean, SD	Interquartile range
1	Personal protective equipment	5.0	4.8, 0.4	0.0*
2	Decontamination	5.0	4.7, 0.5	0.8*
3	Incident management system	5.0	4.7, 0.5	1.0*
4	Mass casualty incidents	5.0	4.6, 0.5	1.0*
5	Basic concepts and nomenclature of disaster medicine	5.0	4.6, 0.5	1.0*
6	Medical response to chemical emergencies	5.0	4.5, 0.6	1.0*
7	Triage	4.5	4.5, 0.6	1.0*
8	Identification, notification, activation, and information collection	4.0	4.4, 0.7	1.0*
9	Medical response to radiation emergencies	4.5	4.3, 0.8	1.0*
10	Medical response to bioterrorism and biological emergencies	4.0	4.3, 0.6	1.0*
11	Mental health	4.0	4.2, 0.7	0.8*
12	Disaster exercises	4.0	4.2, 0.7	1.0*
13	Prehospital disaster management	4.0	4.0, 0.7	0.8*
14	Communication and information management	4.0	4.0, 0.5	0.0*
15	Health consequences of different disasters	4.0	4.0, 0.7	0.8*
16	Fire and burn mass casualty incidents	4.0	4.0, 1.0	1.8
17	Casualty transfer	4.0	3.9, 0.9	0.8*
18	Building collapse and medical response	4.0	3.9, 0.9	1.8
19	Vulnerable populations	4.0	3.8, 0.9	1.0*
20	Blast injuries	4.0	3.8, 1.0	1.8
21	Safety management	4.0	3.7, 0.8	1.0*
22	Casualty identification and tracking	4.0	3.7, 0.8	1.0*
23	Hospital emergency management	4.0	3.7, 0.9	1.0*
24	Isolation and quarantine	4.0	3.7, 0.8	1.0*
25	Legal issues	4.0	3.7, 0.7	1.0*
26	Ethical issues	4.0	3.7, 0.6	1.0*
27	Surge capacity and surge capability	4.0	3.6, 0.9	1.0*
28	Continuity of operation	4.0	3.5, 0.7	1.0*
29	Shelters	3.5	3.5, 0.8	1.0*
30	Hospital evacuation	3.0	3.4, 1.1	1.8
31	Tactical medicine	3.5	3.4, 1.0	1.8
32	Government and non-government organizations	3.5	3.3, 0.9	1.0*
33	Public health issues	3.0	3.3, 1.0	1.8
34	Public information	3.0	3.1, 0.8	1.8
35	Personal and family preparedness	3.0	3.0, 1.0	0.8*
36	Fatality management	3.0	3.0, 0.4	0.0*
37	Recovery	3.0	2.9, 0.7	0.8*
38	Terrorism	3.0	2.9, 1.1	1.8
39	Evidence preservation	3.0	2.9, 1.0	1.0*
40	Resource management	3.0	2.6, 1.0	1.0*
41	National stockpile of pharmaceutical and medical supply	3.0	2.6, 0.8	1.0*
42	Volunteer management	2.0	2.6, 1.1	1.0*

* A high level of agreement after three rounds was defined as an interquartile range of ≤1 and <2 participants changing scores between the final two rounds.

**Table 2:** Disaster medicine core competencies in emergency medicine residency training with high agreement and appropriateness, presented alongside their detailed objectives.

Ranking	Mean score	Competency	Detailed objectives
1	4.8	PPE	Explain the purpose and effects of using PPE. Emphasize the importance of correctly donning and doffing PPE. Describe the training and inspection for PPE. Explain the limitations, risks, and common issues in the use of PPE. Outline the differences in PPE for radiation, biological, and chemical emergencies.
2	4.7	Decontamination	Understand the indications and effects of decontamination. Explain the potential hazards of decontamination procedures for patients and responders. Describe the procedure, equipment, and facilities for emergency decontamination, gross decontamination, and technical procedures. Explain the differences between decontamination of radiation and chemical emergencies. Organize the decontamination post at the incident site, including entry control and casualty flow. Explain the management of waste generated after decontamination.
3	4.7	Incident management system	Explain the concept and importance of the incident management system. Define the Incident Command System (ICS). Describe the advantages and operational principles of applying the ICSto respond to emergencies or disasters, including common terminology, modular organization, unity of command, chain of command, and manageable span of control and unified command. Outline the basic structure of the ICS and the primary tasks of each unit. Explain the similarities and differences between hospital ICS and ICS, as well as their appropriate application. Demonstrate the use of the ICS in exercises or real events. Explain the steps of the planning cycle.
4	4.6	MCIs	Understand the importance of planning for MCIs. Understand the impact of MCIs on regional and local medical resources, as well as their effects on public health. Explain the notification and activation procedures for MCIs in hospitals. Describe common trauma and their management in traumatic MCIs. Explain the potential causes, disease patterns, and management of non-traumatic MCIs.
5	4.6	Basic concepts and nomenclature of disaster medicine	Define and explain the following disaster medicine terms: “emergency,” “disaster,” “multiple casualty incident.” and “MCI.” Explain the following disaster medicine terms: Central Disaster Response System, Disaster Medical Response System, Regional Emergency Medical Operation Center, Incident Command System (ICS), “Emergency Management Program,” “Emergency Operation Plan,” and “Incident Action Plan.” Explain the four phases of emergency management. Explain the “All-hazard” approach in emergency management. Explain the three components of hazard vulnerability analysis: probability of occurrence, impact, and level of preparedness.
6	4.5	Medical response to chemical emergencies	Understand the manifestations of chemical exposure and intoxication of casualties in chemical emergencies. Describe the immediate safety, medical, and other response procedures of first responders in chemical emergencies. Explain the initial identification, notification, and mobilization procedures for chemical emergencies, encompassing both internal and external units. Provide critical information promptly and initiate urgent responses to mitigate potential harm to responders, the environment, and the public. Understand the common chemicals that may lead to emergencies at both regional and national levels, including their characteristics and proper management. Understand the levels of PPE in chemical emergencies and their corresponding indications. Properly don, doff, and dispose of PPEs in chemical emergencies.
7	4.5	Triage	Explain the purpose and indication of triage. Explain the differences between triage during emergencies and disasters and routine triage in emergency departments (Taiwan Triage and Acuity Scale). Triage patients in disasters with varying resources. Explain the differences between prehospital and hospital triage in disasters. Understand the differences in triage for mass gatherings, chemical emergencies, radiation emergencies, and biological emergencies.
8	4.4	Identification, notification, activation, and information collection	Explain the identification procedures of first responders during emergencies, including scenarios, geographical features, potential hazards, and required resources. Explain the notification procedures during emergencies, including recipients, methods, and responsibilities of reporting. Explain the activation procedures during emergencies, including recipients, methods, and responsibilities of activation. Explain the common information collected during emergencies for subsequent analysis and review.
9	4.3	Medical response to radiation emergencies	Explain the basic principles of radiation physics and protection. Explain the resources of hospitals and the government for radiation injuries. Explain the procedures of emergency departments for radiation emergencies prior to the arrival of casualties. Explain the differences in medical response for casualties in chemical emergencies and radiation emergencies. Properly don, doff, and dispose of PPE in radiation emergencies. Understand acute radiation syndrome and explain the classification of casualties based on their initial presentation. Explain the medical treatment for casualties exposed to high-dose radiation within 48 hours.
10	4.3	Medical response to bioterrorism and biological emergencies	Explain the common bioterrorism agents, their modes of dissemination, and possible treatments. Explain the differences between bioterrorism events and general infectious disease outbreaks. Explain the impact of global pandemics, such as COVID-19 or new influenza, on the healthcare system. Explain the response, reporting, and related procedures for suspected cases of unknown emerging infectious diseases or unknown pathogens. Explain the types and differences in PPE for biological emergencies. Describe the optimal PPE for tuberculosis, chickenpox, influenza, Ebola virus, COVID-19, dengue fever, and scabies. Properly don, doff, and dispose of PPE for biological emergencies.
11	4.2	Mental health	Explain the principles of Psychological First Aid. Explain the clinical manifestations of acute stress disorder. Explain the risk factors for post-traumatic stress disorder. Explain the mental health issues in disasters and intervention strategies.
12	4.2	Disaster exercises	Explain the importance of exercises in disaster preparedness. Explain different types of discussion-based exercises (eg, seminars, workshops, tabletop exercises, and games) and operational exercises (eg, drills, functional exercises, and full-scale exercises). Explain the pros and cons of discussion-based exercises and operational exercises. Understand how to design an exercise, do hotwash, and write after-action reports and improvement plans.
13	4.0	Prehospital disaster management	Explain onsite command systems during emergencies or disasters. Explain the setup and functions of medical posts at the scene. Explain the issues and strategies related to casualty referral in MCIs. Explain the coordination and cooperation among various resources and responders at the scene, including police, firefighters, emergency medical technicians, and social workers.
14	4.0	Communication and information management	Explain communication issues during disasters (both external and within hospitals), including assessing the accuracy of information. Understand commonly used communication tools and their pros and cons, as well as alternative communication methods. Understand the importance of maintaining internal and external communication, information exchange, and information security regarding organizational safety. Understand the differences in communication rules within and between organizations.
15	4.0	Health consequences of different disasters	Describe different types of injuries and potential health effects during different phases of different emergencies or disasters: earthquakes, floods, typhoons, cold waves, heatwaves, traffic accidents, chemical emergencies, radiation emergencies, building collapses, explosions, and biological emergencies. Understand the potential impacts of disasters on community healthcare, water, food, and sanitation facilities. Explain common health and medical issues in shelters and their coping strategies.

*PPE*, personal protective equipment; *MCI*, mass casualty incident; *COVID-19*, coronavirus disease 2019.

**Table 3:** Mapping disaster medicine core competencies for emergency medicine residency training in Taiwan to the Accreditation Council for Graduate Medical Education’s six core competencies.

Ranking	DMCC	Six core competencies of medical education					
		PC	MK	PBLI	ICS	P	SBP
1	Personal protective equipment		X				
2	Decontamination	X	X				
3	Incident management system		X				
4	Mass casualty incidents		X				X
5	Basic concepts and nomenclature of disaster medicine		X				
6	Medical response to chemical emergencies	X	X				X
7	Triage	X	X				
8	Identification, notification, activation, and information collection						X
9	Medical response to radiation emergencies	X	X				X
10	Medical response to bioterrorism and biological emergencies	X	X				X
11	Mental health	X	X				
12	Disaster exercises		X				
13	Prehospital disaster management				X		X
14	Communication and information management						X
15	Health consequences of different disasters		X				

*ACGME*, Accreditation Council for Graduate Medical Education; *DMCC*, disaster medicine core competencies; *PC*, patient care; *MK*, medical knowledge; *PBLI*, practice-based learning and improvement; *ICS*, interpersonal and communication skills; *P*, professionalism; *SBP*, systems-based practice.

**Table 4:** Examining core competencies of disaster medicine in emergency medicine residencies in terms of prioritization and consensus in Taiwan vs the United States.

Ranking	Taiwan	The United States^
1	Personal protective equipment*	Patient triage in disasters*
2	Decontamination*	Surge capacity/capability
3	Incident management system	Introduction to disaster medicine/Nomenclature*
4	MCIs*	Blast injuries
5	Basic concepts and nomenclature of disaster medicine*	Hospital disaster mitigation, preparedness, response, and recovery
6	Medical response to chemical emergencies*	Chemical MCI and hospital response*
7	Triage*	Decontamination indications and issues*
8	Identification, notification, activation, and information collection	Trauma MCI*
9	Medical response to radiation emergencies*	Disaster exercises and training*
10	Medical response to bioterrorism and biological emergencies*	Biological agents*
11	Mental health	Personal protective equipment*
12	Disaster exercises*	Radiation MCI and hospital response*
13	Prehospital disaster management	
14	Communication and information management	
15	Health consequences of different disasters	

^ The essential and high-priority disaster medicine educational competencies for emergency medicine residencies in the US.^12^

* The commonalities in EM residency training between Taiwan and the US.

*PPE*, personal protective equipment; *MCI*, mass casualty incident; *COVID-19*, coronavirus disease 2019.
