# Supplementary material for: Disaster Medicine Core Competencies: Comparative Analysis of Emergency Medicine Residency Training in Taiwan and the United States
Source: West J Emerg Med. 2025 Jun 25;26(4):1095–104. doi: 10.5811/westjem.24961 (PMC12342430; doi:10.5811/westjem.24961)
Supplement: Supplementary file 1 [file wjem-26-1095-s001.docx]

**Appendix 1.** Prioritized core competencies of disaster medicine for emergency medicine residencies in Taiwan.

| Ranking | Mean sore | Competency | Detailed objectives |
| --- | --- | --- | --- |
| 1 | 4.8 | PPE | - Explain the purpose and effects of using PPE. - Emphasize the importance of correctly donning and doffing PPE. - Describe the training and inspection for PPE. - Explain the limitations, risks, and common issues in the use of PPE. - Outline the differences in PPE for radiation, biological, and chemical emergencies. |
| 2 | 4.7 | Decontamination | - Understand the indications and effects of decontamination. - Explain the potential hazards of decontamination procedures for patients and responders. - Describe the procedure, equipment, and facilities for emergency decontamination, gross decontamination, and technical procedures. - Explain the differences between decontamination of radiation and chemical emergencies. - Organize the decontamination post at the incident site, including entry control and casualty flow. - Explain the management of waste generated after decontamination. |
| 3 | 4.7 | Incident management system | - Explain the concept and importance of the incident management system. - Define the Incident Command System (ICS). - Describe the advantages and operational principles of applying the Incident Command System to respond to emergencies or disasters, including common terminology, modular organization, unity of command, chain of command, and manageable span of control and unified command. - Outline the basic structure of the ICS and the primary tasks of each unit. - Explain the similarities and differences between Hospital ICS and ICS, as well as their application. - Demonstrate the use of the ICS in exercises or real events. - Explain the steps of the planning cycle. |
| 4 | 4.6 | MCIs | - Understand the importance of planning for MCIs. - Understand the impact of MCIs on regional and local medical resources, as well as their effects on public health. - Explain the notification and activation procedures for MCIs in hospitals. - Describe common trauma and their management in traumatic MCIs. - Explain the potential causes, disease patterns, and management of non-traumatic MCIs. |
| 5 | 4.6 | Basic concepts and nomenclature of disaster medicine | - Define and explain the following disaster medicine terms: “emergency,” “disaster,” “multiple casualty incident,” and “MCI.” - Explain the following disaster medicine terms: Central Disaster Response System, Disaster Medical Response System, Regional Emergency Medical Operation Center, Incident Command System (ICS), “Emergency Management Program,” “Emergency Operation Plan,” and “Incident Action Plan.” - Explain the four phases of emergency management. - Explain the "All-hazard" approach in emergency management. - Explain the three components of hazard vulnerability analysis: probability of occurrence, impact, and level of preparedness. |
| 6 | 4.5 | Medical response to chemical emergencies | - Understand the manifestations of chemical exposure and intoxication of casualties in chemical emergencies. - Describe the immediate safety, medical, and other response procedures of first responders in chemical emergencies. - Explain the initial identification, notification, and mobilization procedures for chemical emergencies, encompassing both internal and external units. - Provide critical information promptly and initiate urgent responses to mitigate potential harm to responders, the environment, and the public. - Understand the common chemicals that may lead to emergencies at both regional and national levels, including their characteristics and proper management. - Understand the levels of PPE in chemical emergencies and their corresponding indications. - Properly don, doff, and dispose of PPEs in chemical emergencies. |
| 7 | 4.5 | Triage | - Explain the purpose and indication of triage. - Explain the differences between triage during emergencies and disasters and routine triage in emergency departments (Taiwan Triage and Acuity Scale). - Triage patients in disasters with varying resources. - Explain the differences between pre-hospital and hospital triage in disasters. - Understand the differences in triage for mass gatherings, chemical emergencies, radiation emergencies, and biological emergencies. |
| 8 | 4.4 | Identification, notification, activation, and information collection | - Explain the identification procedures of first responders during emergencies, including scenarios, geographical features, potential hazards, and required resources. - Explain the notification procedures during emergencies, including recipients, methods, and responsibilities of reporting. - Explain the activation procedures during emergencies, including recipients, methods, and responsibilities of activation. - Explain the common information collected during emergencies for subsequent analysis and review. |
| 9 | 4.3 | Medical response to radiation emergencies | - Explain the basic principles of radiation physics and protection. - Explain the resources of hospitals and the government for radiation injuries. - Explain the procedures of emergency departments for radiation emergencies prior to the arrival of casualties. - Explain the differences in medical response for casualties in chemical emergencies and radiation emergencies. - Properly don, doff, and dispose of PPE in radiation emergencies. - Understand acute radiation syndrome and explain the classification of casualties based on their initial presentation. - Explain the medical treatment for casualties exposed to high-dose radiation within 48 hours. |
| 10 | 4.3 | Medical response in bioterrorism and biological emergencies | - Explain the common bioterrorism agents, their modes of dissemination, and possible treatments. - Explain the differences between bioterrorism events and general infectious disease outbreaks. - Explain the impact of global pandemics, such as COVID-19 or new influenza, on the healthcare system. - Explain the response, reporting, and related procedures for suspected cases of unknown emerging infectious diseases or unknown pathogens. - Explain the types and differences in PPEs for biological emergencies. - Describe the optimal PPEs for tuberculosis, chickenpox, influenza, Ebola virus, COVID-19, dengue fever, and scabies. - Properly don, doff, and dispose of PPEs for biological emergencies. |
| 11 | 4.2 | Mental health | - Explain the principles of Psychological First Aid. - Explain the clinical manifestations of acute stress disorder. - Explain the risk factors for post-traumatic stress disorder. - Explain the mental health issues in disasters and intervention strategies. |
| 12 | 4.2 | Disaster exercises | - Explain the importance of exercises in disaster preparedness. - Explain different types of discussion-based exercises (eg. seminars, workshops, tabletop exercises, and games) and operational exercises (eg, drills, functional exercises, and full-scale exercises). - Explain the pros and cons of discussion-based exercises and operational exercises. - Understand how to design an exercise, do hotwash, and write after-action reports and improvement plans. |
| 13 | 4.0 | Prehospital disaster management | - Explain onsite command systems during emergencies or disasters. - Explain the setup and functions of medical posts at the scene. - Explain the issues and strategies related to casualty referral in MCIs. - Explain the coordination and cooperation among various resources and responders at the scene, including police, firefighters, emergency medical technicians, and social workers. |
| 14 | 4.0 | Communication and information management | - Explain communication issues during disasters (both external and within hospitals), including assessing the accuracy of information. - Understand commonly used communication tools and their pros and cons, as well as alternative communication methods. - Understand the importance of maintaining internal and external communication, information exchange, and information security for organizational safety. - Understand the differences in communication rules within and between organizations. |
| 15 | 4.0 | Health consequences of different disasters | - Describe different types of injuries and potential health effects during different phases of different emergencies or disasters: earthquakes, floods, typhoons, cold waves, heatwaves, traffic accidents, chemical emergencies, radiation emergencies, building collapses, explosions, and biological emergencies. - Understand the potential impacts of disasters on community healthcare, water, food, and sanitation facilities. - Explain common health and medical issues in shelters and their coping strategies. |
| 16 | 4.0 | Fire and burn MCIs | - Address the response strategies for burn MCIs. - Explain the management of burns and inhalation burns. - Describe the management for carbon monoxide poisoning and the indications for hyperbaric oxygen therapy. |
| 17 | 3.9 | Casualty transfer | - Explain the strategies and challenges for the initial and subsequent transfer of casualties, including prioritization. - Describe the preparations, precautions, and limitations for patient transfer, including the acquisition of essential supplies and transportation vehicles. - Understand the transportation vehicles that government agencies may deploy. - Explain the management of transferring selected casualties with special needs in disasters. - Outline the process and key points for interhospital patient transfers. |
| 18 | 3.9 | Building collapse and medical response | - Explain how first responders (non-professional rescue teams) can mark the positions of trapped individuals in buildings and estimate the prognosis of casualties. - Describe the diagnosis, onsite, and hospital treatment of compartment syndrome. - Describe the diagnosis, onsite, and hospital treatment of crush syndrome. - Explain the indications and techniques for field amputation. - Understand the health hazards of dust and its management. - Understand the health hazards of hypothermia and its management. |
| 19 | 3.8 | Vulnerable populations | - List common vulnerable populations in emergencies or disasters, considering aspects such as health and socioeconomic status. - Understand the impact that disasters may have on populations with special medical needs (eg, dialysis treatment, diabetes, chronic respiratory failure). - Explain the challenges and difficulties in dealing with children, the elderly, individuals with dementia, and those with physical or mental disabilities during disasters. - Describe the key points in the emergency response for vulnerable populations. |
| 20 | 3.8 | Blast injuries | - Explain the three main patterns of blast injuries and other possible related injuries (known as the four-level injuries of blasts). - Describe the prehospital and emergency department assessment and management of blast injuries, especially those affecting the ears, intestines, and lungs. - Explain the clinical manifestations of blast injuries and factors related to prognosis. |
| 21 | 3.7 | Safety management | - Explain the importance of safety management during emergencies or disasters. - Describe how safety control procedures may vary based on the nature and severity of the event. - Identify potential vulnerabilities or weaknesses in the safety aspects of hospital buildings or field medical posts. - Describe safety measures to protect casualties and responders during disasters, including entry and exit control procedures. |
| 22 | 3.7 | Casualty identification and tracking | - Explain the purpose and importance of casualty identification and tracking during disasters. - Describe common methods of casualty identification and tracking, along with their pros and cons. - Explain the continuity of casualty tracking from prehospital to hospital settings. - Describe the recording process to ensure the continuity of casualty information. - Explain how to locate family members and assist in reunification. |
| 23 | 3.7 | Hospital emergency management | - Understand the comprehensive emergency management in hospitals. - Explain the three major types of hospital emergencies: mass casualty incidents, environmental emergencies (such as fires and internal disasters), and critical infrastructure failures (such as power outages and oxygen interruptions). - Understand the functions and goals of the crisis management committee. - Describe the structure of the hospital emergency management program, including the main framework of the emergency operations plans during the response to emergencies. - Understand the incident management systems of hospitals. - Understand the resource management and mutual aid agreements with external stakeholders (eg., other healthcare systems, hospitals, vendors, etc.) during emergencies. |
| 24 | 3.7 | Isolation and quarantine | - Explain the concepts of isolation and quarantine. - Describe the roles, purposes, and limitations of isolation and quarantine in public health. - Explain the differences between voluntary and mandatory isolation and quarantine. - Describe the response of emergency departments during the epidemics and pandemics. - Explain the 3-I Tool (Identify-Isolate-Inform) as a response strategy. |
| 25 | 3.7 | Legal issues | - Explain relevant regulations in disaster in Taiwan. - Describe principles and responsibilities in resource allocation during disasters. - Discuss potential changes to healthcare-related regulations during disasters. - Explain possible alterations to medical protocols during disasters. - Explain the issuesof medical errors during disasters. |
| 26 | 3.7 | Ethical issues | - Explain the basic ethical principles in disaster medicine. - List other ethical principles applicable in disasters, serving as references for individuals and groups, and addressing conflicts between different groups. |
| 27 | 3.6 | Surge capacity and surge capability | - Explain the differences between surge capacity and surge capability. - Discuss the relationship between surge capacity, surge capability, and the standards of care in disaster. - Explain the three main core components of surge capacity: Staff, Structure, and Stuff (supplies and equipment). |
| 28 | 3.5 | Continuity of operation | - Explain what continuity of operations in the healthcare system means. - Describe the planning for the continuity of operations in the healthcare system. |
| 29 | 3.5 | Shelters | - Explain the purpose of shelters for the displaced in disasters. - Describe the healthcare needs of residents in shelters. - Explain the healthcare needs of vulnerable populations in shelters. |
| 30 | 3.4 | Hospital evacuation | - Explain the basic principles of hospital evacuation. - Describe the basic principles of evacuating other healthcare facilities (such as nursing homes, long-term care centers, and psychiatric care hospitals). - Explain the healthcare needs of vulnerable populations during evacuations. - Understand the potential resource competition and prioritization of hospital evacuations in the healthcare system. |
| 31 | 3.4 | Tactical medicine | - Explain the specialized training and support of medical staff during conflicts. - Understand the principles of non-military medical operations for civilian hospitals during conflicts. - Describe the collaboration and coordination with military or law enforcement units for responders’ safety during conflicts. |
| 32 | 3.3 | Government and non-government organizations | - Explain the differences between government and non-government organizations, including their goals, capabilities, and limitations. - Describe the differences in disaster response between domestic and international government and non-government organizations. - Explain the three-tier system of disaster medical assistance teams in Taiwan. - Describe the World Health Organization's classification of Emergency Medical Teams. |
| 33 | 3.3 | Public health issues | - Explain the scope of public health during disasters. - Describe what rapid assessment is and how to conduct it. - Describe three common public health issues after disasters and how public health can assist in environmental monitoring. - Describe the unit responsible for public health in the aftermath of disasters. |
| 34 | 3.1 | Public information | - Explain the roles of public information officers and public information centers. - Explain the importance of media management and crisis risk communication. |
| 35 | 3.0 | Personal and family preparedness | - Understand household preparedness for disasters can facilitate effective and efficient responses during disasters. - Describe the elements that should be included in a family disaster response plan, such as communication, individual responsibilities, evacuation, and reunion procedures. - Describe the contents of a home emergency kit. - Describe potential hazards and vulnerabilities in residential communities and items that may require special preparation. - Understand how to access accurate and timely information. |
| 36 | 3.0 | Fatality management | - Explain the goals and importance of managing deceased individuals. - Discuss the importance of fatality management to hospitals during disasters. - Explain how to safely conduct corpse preservation, reporting, and tracking. - Explain why deceased individuals generally do not transmit diseases. |
| 37 | 2.9 | Recovery | - Describe common post-disaster healthcare recovery plans. - Understand the key elements of healthcare facility recovery. |
| 38 | 2.9 | Terrorism | - Explain what terrorism is. - Explain common forms of terrorist attacks. - Describe hospitals’ prevention and mitigation strategies, reporting procedures, and response to terrorist attacks. - Explain the potential risks for first responders during responses, including secondary attacks. - Describe response strategies at the scene of an active shooting incident. - Understand the concept of a dirty bomb. |
| 39 | 2.9 | Evidence preservation | - Explain which emergencies may require evidence preservation and which items are commonly preserved. - Describe methods of evidence preservation. - Explain the cooperation between medical units and law enforcement units in preserving evidence after a terrorist attack. - Describe potential conflicts and solutions between law enforcement agencies and healthcare institutions on personal data protection. |
| 40 | 2.6 | Resource management | - Explain the responsibilities and mechanisms of resource management and mutual support between central and local government. - Describe the relationship between situation analysis and effective resource management. - Explain the local system for obtaining and distributing medical resources (such as disposable items, reusable equipment, medications, and other resources). - Explain how medical routines may adjust when resources are scarce. - Describe how mutual support of resources can be achieved through medical mutual aid agreements. |
| 41 | 2.6 | National stockpile of pharmaceutical and medical supply | - Understand the contents of the national pharmaceutical and medical supply stockpile during peacetime and conflicts. - Describe the pharmaceutical and medical supply stockpile within hospitals during conflicts. |
| 42 | 2.6 | Volunteer management | - Explain the possible sources, types, and backgrounds of volunteers. - Describe the management and job assignment of volunteers and the potential impact on response actions. - Explain the protection and welfare of volunteers. - Explain the importance of emergency-certified volunteers. - Describe the training and management for volunteers in the medical profession. |

*PPE*, personal protective equipment; *MCI*, mass casualty incident; *COVID-19*, coronavirus disease 2019.

**Appendix 2.** The match between the disaster medicine core competencies for emergency medicine residencies in Taiwan and the six core competencies of medical education of the ACGME.

| Ranking | DMCC | Six core competencies of medical education | | | | | |
| --- | --- | --- | --- | --- | --- | --- | --- |
|  |  | PC | MK | PBLI | ICS | P | SBP |
| 1 | Personal protective equipment |  | X |  |  |  |  |
| 2 | Decontamination | X | X |  |  |  |  |
| 3 | Incident management system |  | X |  |  |  |  |
| 4 | Mass casualty incidents |  | X |  |  |  | X |
| 5 | Basic concepts and nomenclature of disaster medicine |  | X |  |  |  |  |
| 6 | Medical response to chemical emergencies | X | X |  |  |  | X |
| 7 | Triage | X | X |  |  |  |  |
| 8 | Identification, notification, activation, and information collection |  |  |  |  |  | X |
| 9 | Medical response to radiation emergencies | X | X |  |  |  | X |
| 10 | Medical response to bioterrorism and biological emergencies | X | X |  |  |  | X |
| 11 | Mental health | X | X |  |  |  |  |
| 12 | Disaster exercises |  | X |  |  |  |  |
| 13 | Prehospital disaster management |  |  |  | X |  | X |
| 14 | Communication and information management |  |  |  |  |  | X |
| 15 | Health consequences of different disasters |  | X |  |  |  |  |
| 16 | Fire and burn mass casualty incidents | X | X |  |  |  |  |
| 17 | Casualty transfer | X | X |  |  |  | X |
| 18 | Building collapse and medical response | X | X |  |  |  |  |
| 19 | Vulnerable populations | X | X |  |  | X |  |
| 20 | Blast injuries | X | X |  |  |  |  |
| 21 | Safety management |  |  |  |  |  | X |
| 22 | Casualty identification and tracking |  | X |  |  |  | X |
| 23 | Hospital emergency management |  |  |  |  |  | X |
| 24 | Isolation and quarantine |  | X |  |  |  |  |
| 25 | Legal issues |  |  |  |  | X |  |
| 26 | Ethical issues |  |  |  |  | X |  |
| 27 | Surge capacity and surge capability |  |  |  |  |  |  |
| 28 | Continuity of operation |  | X |  |  |  |  |
| 29 | Shelters | X | X |  |  |  |  |
| 30 | Hospital evacuation | X |  |  |  |  | X |
| 31 | Tactical medicine | X | X |  |  |  | X |
| 32 | Government and non-government organizations |  | X |  |  |  | X |
| 33 | Public health issues |  | X |  |  |  | X |
| 34 | Public information |  |  |  | X |  |  |
| 35 | Personal and family preparedness |  | X |  |  |  |  |
| 36 | Fatality management |  | X |  |  |  | X |
| 37 | Recovery |  | X |  |  |  |  |
| 38 | Terrorism |  |  |  |  |  |  |
| 39 | Evidence preservation |  |  |  |  |  | X |
| 40 | Resource management |  |  |  |  |  | X |
| 41 | National stockpile of pharmaceutical and medical supply |  |  |  |  |  | X |
| 42 | Volunteer management |  |  |  |  |  | X |

^^^ *ACGME*, Accreditation Council for Graduate Medical Education; *DMCC*, disaster medicine core competencies; *PC*, patient care; *MK*, medical knowledge; *PBLI*, practice-based learning and improvement; *ICS*, interpersonal and communication skills; *P*, professionalism; *SBP*, systems-based practice.
